# Decreased dengue cases attributable to the effect of COVID-19 in Guangzhou in 2020

**DOI:** 10.1371/journal.pntd.0009441

**Published:** 2021-05-26

**Authors:** Liyun Jiang, Yuan Liu, Wenzhe Su, Wenhui Liu, Zhicong Yang

**Affiliations:** 1 Virology Department, Guangzhou Centre for Disease Control and Prevention, Baiyunqu Qidelu 1, Guangdong, China; 2 Pesticide and Disinfection Department, Guangzhou Centre for Disease Control and Prevention, Baiyunqu Qidelu 1, Guangdong, China; 3 Epidemiology Department, Guangzhou Centre for Disease Control and Prevention, Baiyunqu Qidelu 1, Guangdong, China; University of Hong Kong, HONG KONG

## Abstract

The dengue fever epidemic in Guangzhou may have been affected by the Coronavirus Disease 2019 (COVID-19) pandemic. The number of dengue cases dropped drastically in 2020, and there have been only 2 local cases, suggesting that dengue has not become endemic in Guangzhou.

Guangzhou is located on the southeast coast of China and is the country’s third largest city. Since 1978, outbreaks of dengue fever have occurred intermittently in this city. In the past decade, the number of reported dengue cases reached more than 1,000 in 2013, 2014, 2018, and 2019, with 37,385 cases reported in 2014 alone. Therefore, dengue fever is a major public health concern in Guangzhou, and there is a continuing argument that it is endemic in Guangzhou [1–3].

The numbers of dengue cases from 2017 to 2020 are shown in Table 1. In 2020, the total and local case numbers dropped dramatically compared to the previous years. With a high proportion of imported cases (*n =* 32, 94.12%), the proportion of local cases (*n* = 2, 5.88%) was considerably low in 2020. All the prevention and control strategies for dengue, including issuing public education messages, preventing further mosquito bites in patients, cleaning vector breeding sites, and using pesticides, were similar during these years. Additionally, dengue, as a mosquito-borne viral infectious disease, is closely related to mosquito density. The mosquito ovitrap index (MOI), which is the proportion of positive mosquito ovitraps, is usually used to indicate mosquito density. The MOI in 2017, 2018, and 2019 was 7.073 ± 1.016, 9.657 ± 1.307, and 8.464 ± 0.961, respectively. The average MOI was 8.398 ± 0.648 from 2017 to 2019 in Guangzhou. The MOI in 2020 was 7.135 ± 0.786, which remained at the median risk level. Therefore, the abnormal decline in dengue cases could not be attributed to the change in mosquito density in 2020.

**Table 1:** Numbers and percentages of dengue cases from 2017 to 2019.

Year	2017	2018	2019	2020
Total cases	944	1,295	1,655	34
Imported cases(percentage)	69 (7.31%)	96 (7.41%)	270 (16.31%)	32 (94.12%)
Local cases (percentage)	875 (92.69%)	1,199 (92.59%)	1,385 (83.69%)	2 (5.88%)

In 2020, the 14-day quarantine in a designated hotel for international travelers to curb the spread of Coronavirus Disease 2019 (COVID-19) was an important public health intervention. People had to remain indoors except for medical care needs. All imported dengue cases were identified during their quarantine periods. No secondary case related to the imported cases was reported. This may be because *Aedes albopictus*, which is the major vector of dengue in Guangzhou, bites aggressively during the day outdoors. The chance of being bitten by *A*. *albopictus* was reduced by staying all day indoors. Moreover, some research revealed that viremia occurred 6 to 18 hours before symptoms appeared and lasted as long as 12 days [4]. After the 14-day quarantine, viremia had almost subsided. Therefore, imported dengue cases were unlikely to be transmitted. The impact of imported dengue cases was limited by the quarantine, which provided a rare opportunity to identify the local epidemic.

The epidemiology investigation showed that the 2 local cases, who were living in the same building, had no travel history outside Guangzhou in 2020 and had symptoms successively. Two dengue virus serotype 2 (DENV-2) strains were isolated from them. The envelope gene sequences were obtained and deposited in GenBank under accession numbers MW295818 and MW345921. Reference sequences, which were downloaded from GenBank, and sequences of Guangzhou strains identified in the previous years, were used to construct a phylogenetic tree. The 2 isolated strains in 2020 were identical. The tree (Fig 1) shows that the 2 strains belonged to the Malaysia/Indian subcontinent genotype, which was the prevailing genotype in Guangzhou [5]. However, they were neither identical with nor derived from the Guangzhou strains obtained from the previous years. Using the Basic Local Alignment Search Tool in GenBank, the 2 strains were found to be highly similar to those identified in Zhejiang (China), Singapore, and Guangdong (China) in 2017. These results imply that the local cases may be secondary to some undiscovered cases imported from other cities in China, as no restriction and quarantine was imposed for domestic travels.

**Fig 1:**
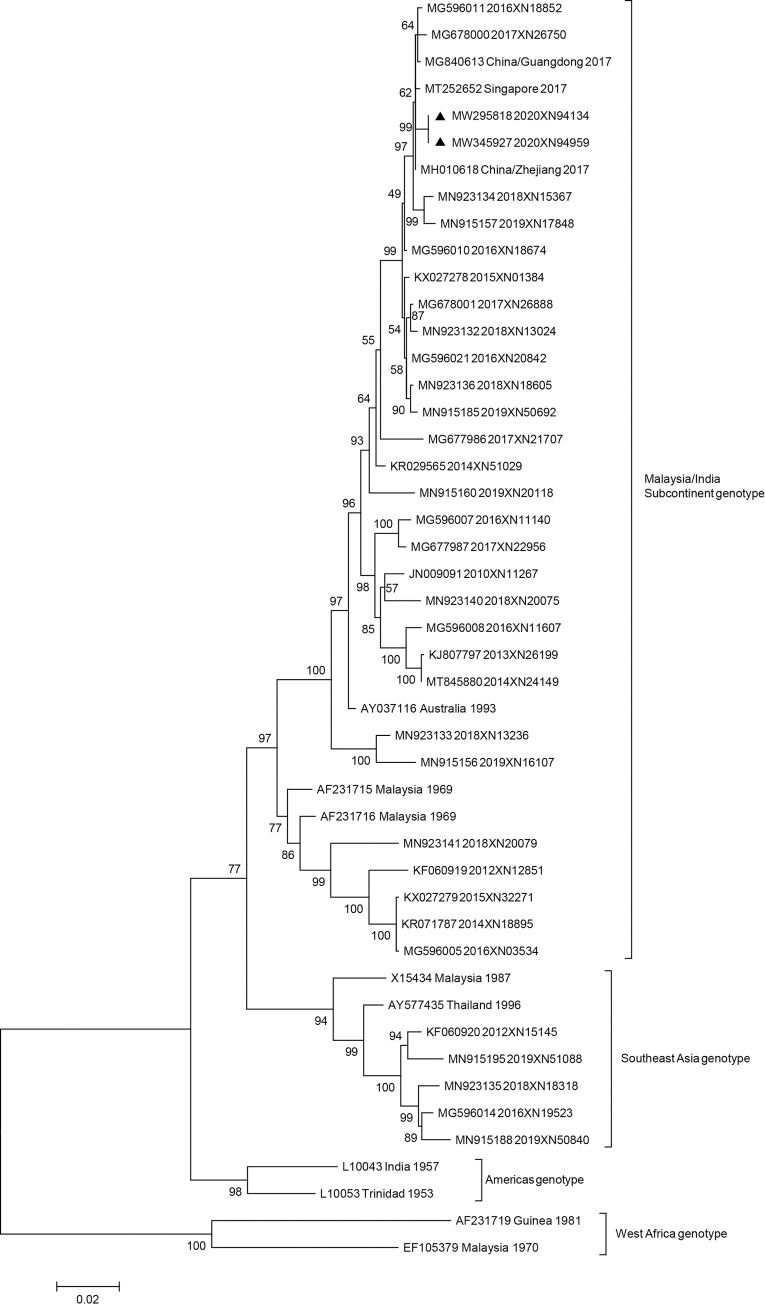
Maximum-likelihood phylogenetic tree shows the evolutionary relationships of DENV-2 detected in the sera of 2 local cases along with 45 other sequences. The reference sequences are named using the GenBank accession number, country, and year. The sequences of strains isolated in Guangzhou are named using the GenBank accession number, year, and our lab number. Bootstrap support values are shown in the notes. Strains isolated in 2020 are indicated with a black triangle.

When the impact of imported dengue cases was limited by quarantine, dengue did not spread in Guangzhou during 2020, with the MOI still at median risk level and without any changes in the prevention and control strategies. Moreover, serotype 1 had been prevalent in Guangzhou since 2011 [6,7]. However, there was no local infection of serotype 1 detected in 2020. These observations may provide further evidence that dengue fever is not endemic in Guangzhou.

In conclusion, the number of dengue cases decreased during the COVID-19 epidemic in Guangzhou in 2020. Thus, we believe that dengue fever is not endemic in Guangzhou.

## Ethics statement

The Ethics Committee of Guangzhou Centre for Disease Control and Prevention approved the present study. Written informed consent was obtained from all participants in the study. Written formal consent was obtained from the parent/guardian when child participants (younger than 18) were involved. All procedures involved in this work complied with the ethical standards of the relevant national and institutional committees on human experimentation and with the Declaration of Helsinki (2008 amendment).
